# Association of Interleukin-1 gene clusters polymorphisms with primary open-angle glaucoma: a meta-analysis

**DOI:** 10.1186/s12886-017-0616-y

**Published:** 2017-11-28

**Authors:** Junhua Li, Yifan Feng, Mi Sun Sung, Tae Hee Lee, Sang Woo Park

**Affiliations:** 1 Department of Ophthalmology, Chonnam National University Medical School and Hospital, 42 Jebongro, Gwang-ju, 61469 Republic of Korea; 2 Department of Ophthalmology, Zhongshan Hospital, Fudan University, Shanghai, 200032 People’s Republic of China

**Keywords:** Interleukin-1, Polymorphism, Primary open-angle glaucoma, Meta-analysis

## Abstract

**Background:**

Previous studies have associated the Interleukin-1 (*IL-1*) gene clusters polymorphisms with the risk of primary open-angle glaucoma (POAG). However, the results were not consistent. Here, we performed a meta-analysis to evaluate the role of *IL-1* gene clusters polymorphisms in POAG susceptibility.

**Methods:**

PubMed, EMBASE and Cochrane Library (up to July 15, 2017) were searched by two independent investigators. All case-control studies investigating the association between single-nucleotide polymorphisms (SNPs) of *IL-1* gene clusters and POAG risk were included. Odds ratios (ORs) with 95% confidence intervals (CIs) were calculated for quantifying the strength of association that has been involved in at least two studies.

**Results:**

Five studies on *IL-1β* rs16944 (c. -511C > T) (1053 cases and 986 controls), 4 studies on *IL-1α* rs1800587 (c. -889C > T) (822 cases and 714 controls), and 4 studies on *IL-1β* rs1143634 (c. +3953C > T) (798 cases and 730 controls) were included. The results suggest that all three SNPs were not associated with POAG risk. Stratification analyses indicated that the rs1143634 has a suggestive associated with high tension glaucoma (HTG) under dominant (*P* = 0.03), heterozygote (*P* = 0.04) and allelic models (*P* = 0.02), however, the weak association was nullified after Bonferroni adjustments for multiple tests.

**Conclusions:**

Based on current meta-analysis, we indicated that there is lack of association between the three SNPs of *IL-1* and POAG. However, this conclusion should be interpreted with caution and further well designed studies with large sample-size are required to validate the conclusion as low statistical powers.

**Electronic supplementary material:**

The online version of this article (doi: 10.1186/s12886-017-0616-y) contains supplementary material, which is available to authorized users.

## Background

Glaucoma is the second largest cause of bilateral blindness worldwide that affects approximately 60 million people [1]. Primary open-angle glaucoma (POAG), the major type of glaucoma, is characterized by progressive damage of the optic nerve and retinal ganglion cells (RGC), and subsequent irrevocable vision loss [2]. Although the etiology is not entirely clear, genetic factors are thought to be a potential risk to POAG patients, including both those with normal and elevated intraocular pressure (IOP). Recently, the relationship between genetic polymorphisms of the immune system and were not perceived as being related to glaucoma, however, their relationship has been highly suspected and investigated recently [3].

Interleukin-1 (*IL-1*), an important mediator of inflammation, has been suggested to play a crucial role in neuro-degeneration such as Alzheimer’s disease [4]. In view of the potential similarities in cellular mechanism leading to neuro-degenerative disorder between glaucoma and Alzheimer’s disease (AD), some investigators speculated that the polymorphism in *IL-1* gene clusters may be a genetic predisposing factor for glaucoma. It has been reported that *IL-1* was expressed endogenously in trabecular meshwork (MT) cells that controls a stress response specific to the aqueous outflow pathway and confers a protective response against glaucoma [5]. Moreover, experiments in vivo showed that *IL-1* promotes glutamate uptake by Müller cells and increases the number of surviving of RGCs [6].

Recently, several single nucleotide polymorphisms (SNPs) in the IL-1 gene cluster [i.e. *IL-1β* rs16944 (c. -511C > T), *IL-1α* rs1800587 (c. -889C > T) and *IL-1β* rs1143634 (c. +3953C > T)] have been investigated for association with POAG [7–14]. However, the results remain controversial due to some studies identified there was an association between *IL-1* gene SPN and POA while others failed. Furthermore, to our knowledge, no relevant genome-wide association study (GWAS) or meta-analysis has been published on this subject. In the current study, we conducted a systematic review and meta-analysis to summarize the contradictory results from these relevant studies and to clarify the relationship between *IL-1* cluster polymorphisms and POAG.

## Methods

This meta-analysis was followed the the Preferred Reporting Items for Systematic Review and Meta-Analyses (PRISMA) statement [15].

### Literature search

To obtain eligible literature, a comprehensive search without a language restriction was conducted in the PubMed, EMBASE and Cochrane Library covering publications up to July 15, 2017, by using of the following keywords: (“glaucoma” or “open angle” or “POAG” or “intraocular hypertension”) AND (“interleukin-1” or “*IL-1*” or “interleukin 1”) AND (“polymorphism” or “SNP” or “single nucleotide polymorphism” or “variation” or “mutation”) (Additional file 1). The titles, abstracts, and full texts were screened by two independent investigators (L.J.H., F.Y.F.) to determine inclusion and disagreements were resolved by discussion between all authors until a consensus was determined. Review articles and bibliographies of other relevant studies were searched manually to identify additional eligible studies.

### Inclusion and exclusion criteria

Literature selection had to meet the the following criteria: (1) case-control or cohort studies on the relationship of the SNPs of *IL-1* with POAG; (2) all patients in the eligible studies meeting the diagnostic criteria for POAG; (3) had sufficient information for estimating odds ratios (ORs) with corresponding 95% confidence interval (CI). The exclusion criteria were: (1) case only studies; (2) abstract, case report and review papers; (3) repeated or overlapped publication. Only the most recent or complete study was used in this meta-analysis in case of the same patient population was included in several publications.

### Quality assessment

The methodological quality of eligible studies was evaluated independently by two authors (J.H.L. and Y.F.F.), according to a modified version of the Newcastle–Ottawa scale (NOS) for genetic association studies [16] (Additional file 2). NOS quality scores ranged between 0 and 9 stars. Studies with a score of five stars or greater were considered high quality.

### Data extraction

According to the PRISMA guidance, the necessary information from eligible studies was extracted independently by two reviewers (L.J.H. and S.M.S.) using a standardized form. The following data was retrieved: surname of first author, publication date, region, ethnicity, number of cases and controls, age, subtypes of POAG, genotyping method and genotypes frequency, evidence of Hardy-Weinberg equilibrium (HWE) in control group, etc. When the allele or genotype counts were not given specially in some articles, they were calculated from the frequencies and then rounded to the nearest integer. Disagreements were resolved by discussion between all authors and subsequent consensus.

### Statistical analysis

Statistical analysis was undertaken using RevMan software (version 5.0; Cochrane Collaboration, Oxford, United Kingdom) and STATA 11.0 software (Stata Corporation, Texas, USA). The strength of association between *IL-1* SNPs and POAG was estimated by ORs with 95% confidence intervals (CIs). The pooled ORs were performed for the allele model (T versus C), homozygote model (CC versus TT), heterozygote model (TC versus CC), dominant model (TT + CT versus CC) and recessive model (TT versus CT + CC), respectively.

I^2^value was applied to evaluate the between-study heterogeneity, with <25%, 25%–50%, and >50% to represent low, moderate, and high degree of heterogeneity, respectively [17]. In addition, a Q-statistic test was conducted and the heterogeneity was considered significant at *P* < 0.10. The summary OR and 95% CI for each polymorphism were pooled by using the fixed-effect model (Mantel-Haenszel) when no significant heterogeneity was observed among studies. Otherwise, the random effect model (DerSimonian and Laird) was adopted.

In association analysis, a pooled *P* value of less than 0.05 was considered as suggestive evidence for a genetic association. Aim to control false positive error rate, the Bonferroni method was used to adjust for multiple comparisons [18]. We performed 20 times comparisons for *IL-1β* rs16944, 15 times comparisons for *IL-1α* rs1800587 and *IL-1β* rs1143634 in POAG respectively, therefore, the *P* values that were less than 0.05/20 in *IL-1β* rs16944 and 0.05/15 in *IL-1α* rs1800587 and *IL-1β* rs1143634 showed significance.

Power analysis regarding the association of each *IL-1* SNP with POAG was performed using the statistical software Power and Sample Size Calculation (PS) version 3.1.2 (http://biostat.mc.vanderbilt.edu/wiki/Main/PowerSampleSize) and a value greater than 0.8 meant high statistical power. The HWE was assessed by Fisher’s exact test. Subgroup analyses were performed based on ethnicity, as well as type of POAG including high tension glaucoma (HTG) (IOP > 21 mmHg) and non-HTG (IOP < 21 mmHg, with or without a strict diurnal testing). Sensitivity analysis was performed to assess the stability of individual studies, and the potential of publication bias was assessed by Begg’s funnel plot [19] and further evaluated by Egger’s linear regression test [20].

## Results

### Eligible studies

**Fig. 1:**
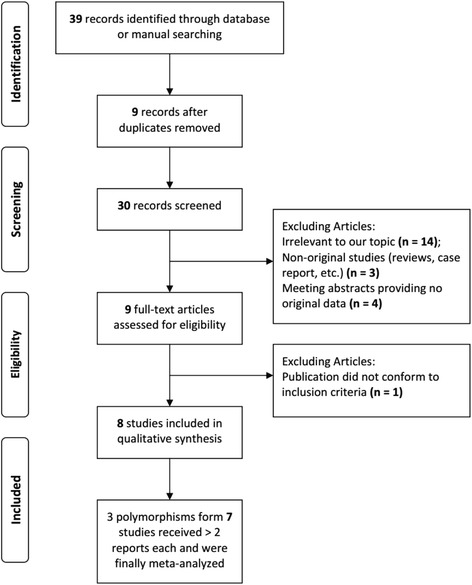
A systematic flow chart detailing the selection of study

The flow chart for the study selection is summarized in Fig. 1. The initial search yielded 39 articles and 9 duplicate publications were excluded **(**Additional file 1 **)**. Based on the title and content of the abstract, 22 studies were further excluded. Nine articles with full-texts were further reviewed and 1 publication was excluded because it did not conform to our inclusion criteria. Thus, 8 case-control studies [7–14] were finally included in this meta-analysis and the main characteristics of each study are listed in Additional file 3: Table S1. The publication year of the included studies ranged from 2003 to 2016. All of the 8 studies had scores ≥5* and the mean score was 5.6*. The quality of these studies is summarized in Additional file 4: Table S2. In total, 64 SNPs have been investigated at least once in these 8 studies, among which 3 SNPs (*IL-1β* rs16944, *IL-1β* rs1143634, *IL-1α* rs1800587) were tested in at least 2 studies and included in the present meta-analysis. In all studies, the distributions of genotypes of all SNP in the control groups were consistent with HWE (Table 1).

**Table 1:** Characteristics of the studies included in the meta-analysis

Authors/Year	Country	Ethnicity	Genotyping method	Type of POAG^a^	Subjects (n)		MAF (%)		Genotypes for Cases			Genotypes for Controls			Test for HWE	
					Cases	Controls	Cases	Controls	CC	CT	TT	CC	TC	TT	X^2^	P
*IL-1β* rs16944																
Lin et al./2003 [13]	Taiwan	Asian	PCR-SSP	All	58	105	43.1	49.5	18	30	10	28	50	27	0.24	0.63
How et al./2007 [9]	Singapore	Asian	PCR-DS	All HTG non-HTG	194 100 94	79	46.4 42.0 51.1	43.0	56 35 21	96 46 50	42 19 23	24	42	13	0.56	0.45
Wang et al./2007 [10]	Taiwan	Asian	PCR-RFLP	non-HTG	231	245	46.3	49.6	70	108	53	61	125	59	0.10	0.75
Markiewicz et al./2013 [7]	Poland	Caucasian	PCR-RFLP	All	255	256	39.4	30.6	93	123	39	118	119	19	2.22	0.14
Mookherjee et al./2010 [8]	India	Asian	PCR-RFLP	All HTG non-HTG	315 116 199	301	56.2 55.6 56.6	60.6	61 23 38	154 57 97	100 36 64	47	143	111	0.007	0.93
*IL-1α* rs1800587																
How et al./2007 [9]	Singapore	Asian	PCR-DS	All HTG non-HTG	189 100 89	79	8.7 7.0 10.7	10.8	158 87 71	29 12 17	2 1 1	64	13	2	1.62	0.20
Wang et al./2007 [11]	Taiwan	Asian	PCR-RFLP	non-HTG	162	167	14.5	13.2	118	41	3	125	40	2	0.37	0.54
Wang et al./2006 [12]	Taiwan	Asian	PCR-RFLP	HTG	156	167	21.2	13.2	98	50	8	125	40	2	0.37	0.54
Mookherjee et al./2010 [8]	India	Asian	PCR-RFLP	All HTG non-HTG	315 116 199	301	28.9 34.5 25.6	32.2	160 50 110	128 52 76	27 14 13	138	132	31	0.005	0.95
*IL-1β* rs1143634																
How et al./2007 [9]	Singapore	Asian	PCR-DS	All HTG non-HTG	194 100 94	79	1.8 3.0 0.5	1.2	187 94 93	7 6 1	0 0 0	77	2	0	0.01	0.91
Lin et al./2003 [13]	Taiwan	Asian	PCR-SSP	All	58	105	6.0	1.5	51	7	0	102	3	0	0.02	0.88
Wang et al./2007 [10]	Taiwan	Asian	PCR-RFLP	non-HTG	231	245	3.9	4.1	214	16	1	226	18	1	0.93	0.33
Mookherjee et al./2010 [8]	India	Asian	PCR-RFLP	All HTG non-HTG	315 116 199	301	14.6 16.8 13.3	11.5	230 80 150	78 33 45	7 3 4	236	61	4	0.0007	0.98

*HTG* high tension glaucoma, *PCR* polymerase chain reaction, *DS* direct sequencing, *RFLP* restriction fragment length polymorphism, *SSP* sequence-specific primer, *MAF* minor allele frequency

^a^POAG patients were subdivided to two groups: HTG (IOP > 21 mmHg) and non-HTG (IOP < 21 mmHg, with or without a strict diurnal testing)

### Meta-analysis

#### Association between SNP rs16944 of *IL-1β* gene and POAG

**Fig. 2:**
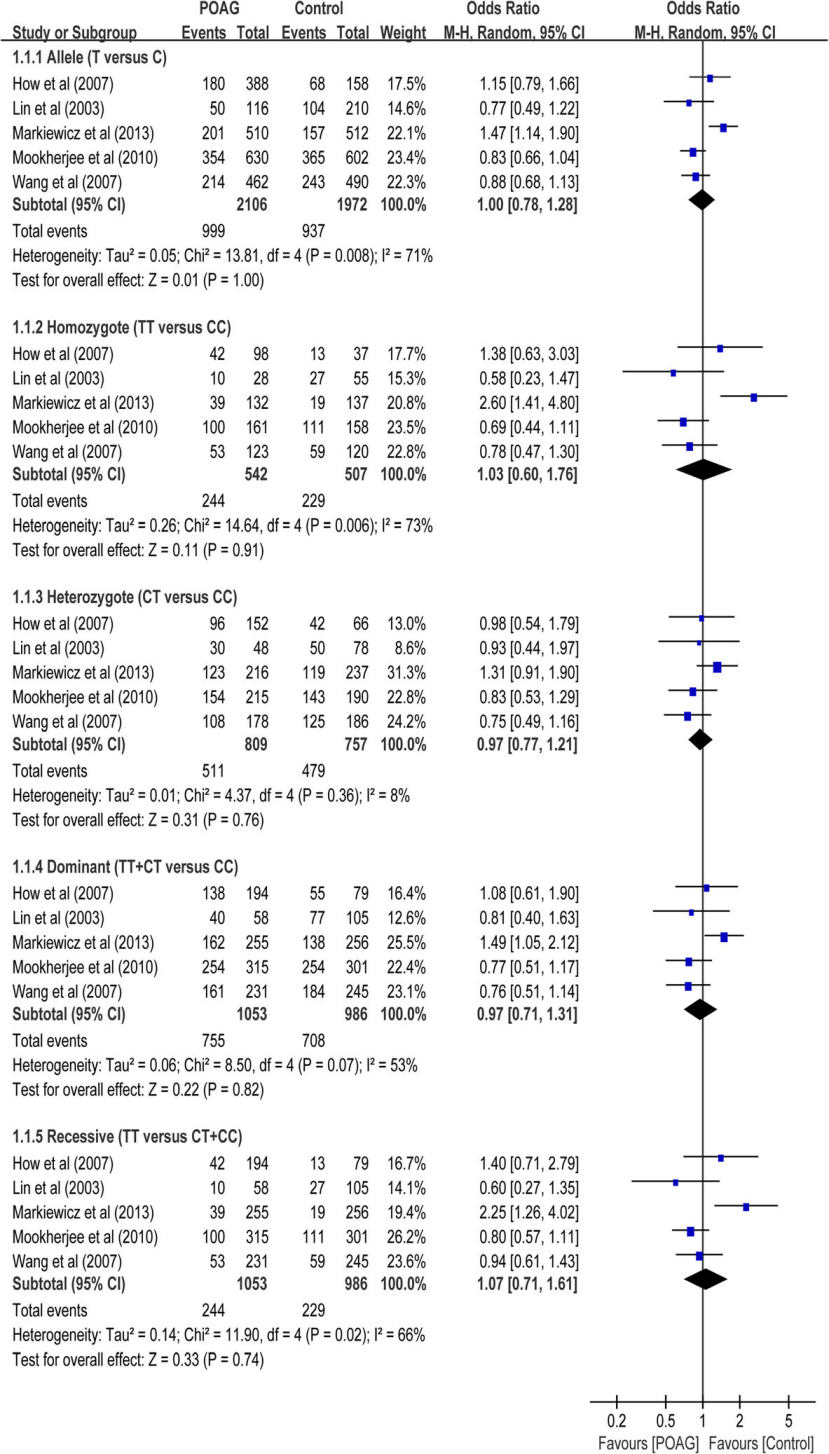
Forest plot for included studies examining the association between the *IL-1β* rs16944 and POAG risk. Abbreviations: POAG, primary open-angle glaucoma; Chi^2^, Chi-square statistic; CI, confidence interval; df, degrees of freedom; I^2^, I-square heterogeneity statistic; IV, inverse variance; Z, Z-statistic

SNP rs16944 was assessed in 5 case-control studies with a total of 1053 POAG cases and 986 controls (Table 1). No association between this SNP and POAG risk was found in the overall populations in dominant (OR = 0.97, 95% CI 0.71 to 1.31, *P* = 0.83), recessive (OR = 1.07, 95% CI 0.71 to 1.61, *P* = 0.74), homozygote (OR = 1.03, 95% CI 0.60 to 1.76, *P* = 0.91), heterozygote (OR = 0.97, 95% CI 0.79 to 1.20, *P* = 0.91) and allelic comparison (OR = 1.00, 95% CI 0.78 to 1.28, *P* = 1.00) models (Table 2; Fig. 2). In subgroup analysis stratified by type of POAG, the meta-analyses indicated no significant association of this SNP with POAG in any of the genetic models in patients with HTG and non-HTG, respectively. In subgroup analysis stratified by ethnicity, the meta-analyses indicated no significant association of this SNP with POAG in any of the genetic models in Asians.

**Table 2:** Stratified analyses between the *IL-1* gene polymorphisms and risk of POAG

Subgroup	No. of studies	No. of patients		Allele model		Homozygote model		Heterozygote model		Dominant model		Recessive model		Power calculation^a^z
		Cases	Controls	OR (95% CI)	P	OR (95% CI)	P	OR (95% CI)	P	OR (95% CI)	P	OR (95% CI)	P	
*IL-1β* rs16944														
Overall	5	1053	986	1.00 [0.78, 1.28]	1.00	1.03 [0.60, 1.76]	0.91	0.97 [0.79, 1.20]	0.77	0.97 [0.71, 1.31]	0.83	1.07 [0.71, 1.61]	0.74	5.1%
Asian	4	798	730	0.88 [0.76, 1.02]	0.09	0.79 [0.58, 1.06]	0.11	0.84 [0.65, 1.08]	0.18	0.82 [0.64, 1.05]	0.11	0.87 [0.69, 1.10]	0.26	42.2%
HTG	2	216	380	0.86 [0.67, 1.10]	0.24	0.76 [0.46, 1.27]	0.30	0.79 [0.51, 1.22]	0.28	0.78 [0.51, 1.18]	0.23	0.86 [0.58, 1.27]	0.45	23.8%
non-HTG	3	524	625	0.96 [0.75, 1.23]	0.73	0.93 [0.56, 1.54]	0.77	0.76 [0.46, 1.27]	0.34	0.87 [0.65, 1.14]	0.31	0.94 [0.72, 1.22]	0.63	7.7%
*IL-1α* rs1800587														
Overall	4	822	714	1.08 [0.75, 1.55]	0.67	1.19 [0.44, 3.17]	0.73	1.02 [0.81, 1.29]	0.84	0.99 [0.64, 1.54]	0.98	1.01 [0.64, 1.61]	0.96	14.1%
HTG	3	372	547	1.15 [0.71, 1.86]	0.57	1.51 [0.83, 2.73]	0.18	1.19 [0.87, 1.62]	0.27	1.17 [0.71, 1.91]	0.54	1.41 [0.80, 2.50]	0.23	24.7%
non-HTG	3	450	547	1.84 [0.67, 1.05]	0.12	0.59 [0.32, 1.10]	0.10	0.87 [0.66, 1.16]	0.34	0.87 [0.62, 1.23]	0.44	0.66 [0.36, 1.21]	0.18	35.7%
*IL-1β* rs1143634														
Overall	4	798	730	1.32 [0.99, 1.75]	0.06	1.64 [0.53, 5.11]	0.39	1.32 [0.96, 1.81]	0.09	1.34 [0.98, 1.82]	0.07	1.56 [0.50, 4.84]	0.44	51.1%
HTG	2	216	380	1.61 [1.06, 2.42]	0.02	2.21 [0.48, 10.10]	0.31	1.65 [1.03, 2.65]	0.04	1.69 [1.07, 2.67]	0.03	1.97 [0.43, 8.95]	0.38	73.3%
non-HTG	3	524	625	1.10 [0.79, 1.53]	0.57	1.45 [0.42, 5.07]	0.56	1.07 [0.74, 1.54]	0.72	1.09 [0.76, 1.55]	0.64	1.42 [0.41, 4.93]	0.59	9.4%

*HTG* high tension glaucoma

^a^Power calculations were performed for “Allele models”

#### Association between SNP rs1800587 of *IL-1α* gene and POAG

**Fig. 3:**
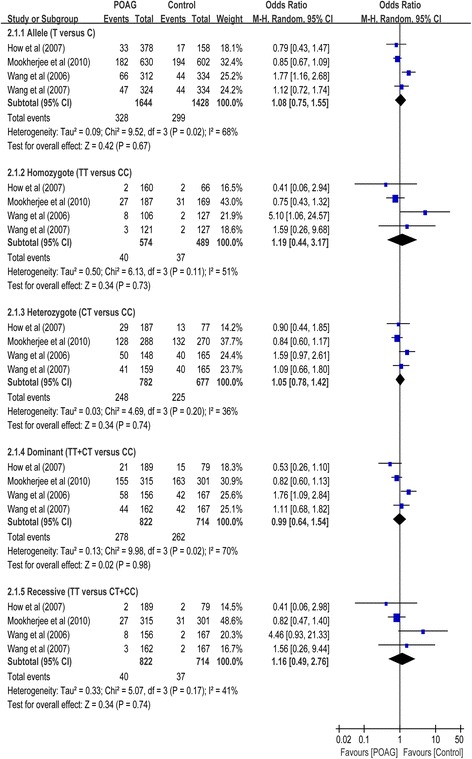
Forest plot for included studies examining the association between the *IL-1α* rs1800587 and POAG risk. Abbreviations: POAG, primary open-angle glaucoma; Chi^2^, Chi-square statistic; CI, confidence interval; df, degrees of freedom; I^2^, I-square heterogeneity statistic; IV, inverse variance; Z, Z-statistic

Four case-control studies had investigated the relationship between SNP rs1800587 and susceptibility to POAG with a total of 822 cases and 714 control subjects (Table 1). No association between this SNP and POAG risk was found in the overall populations in dominant (OR = 0.99, 95% CI 0.64 to 1.54, *P* = 0.98), recessive (OR = 1.01, 95% CI 0.64 to 1.61, *P* = 0.96), homozygote (OR = 1.19, 95% CI 0.44 to 3.17, *P* = 0.73), heterozygote (OR = 1.02, 95% CI 0.81 to 1.29, *P* = 0.84) and allelic comparison (OR = 1.08, 95% CI 0.75 to 1.55, *P* = 0.67) models (Table 2; Fig. 3). In subgroup analysis stratified by type of POAG, the meta-analyses indicated no significant association of this SNP with POAG in any of the genetic models in patients with HTG and non-NTG, respectively **(**Table 2 **)**.

#### Association between SNP rs1143634 of *IL-1β* gene and POAG

**Fig. 4:**
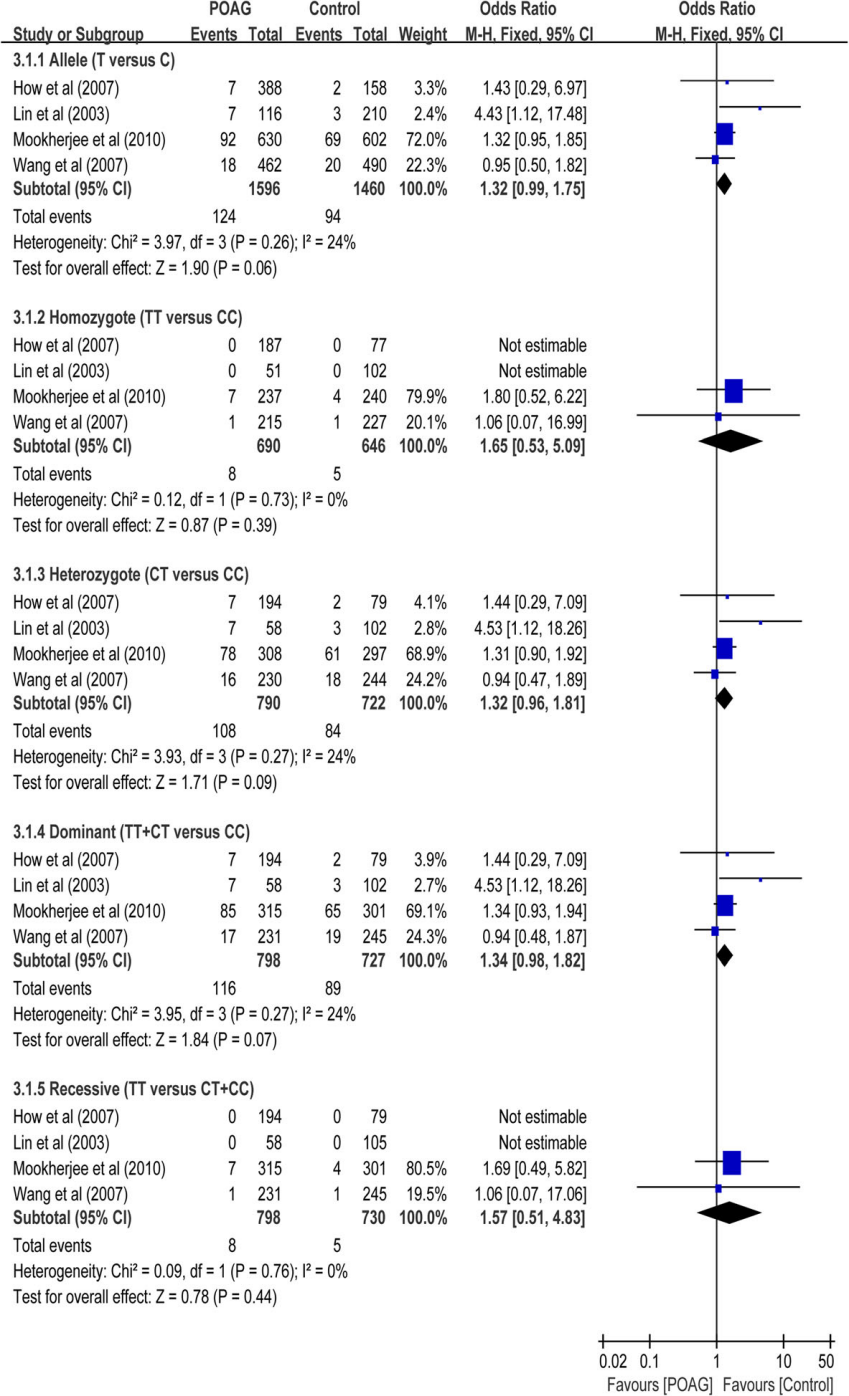
Forest plot for included studies examining the association between the *IL-1β* rs1143634 and POAG risk. Abbreviations: POAG, primary open-angle glaucoma; Chi^2^, Chi-square statistic; CI, confidence interval; df, degrees of freedom; I^2^, I-square heterogeneity statistic; IV, inverse variance; Z, Z-statistic

Three case-control studies had investigated the relationship between SNP rs1143634 and susceptibility to POAG with a total of 798 cases and 730 control subjects (Table 1). No association between this SNP and POAG risk was found in the overall populations in dominant (OR = 1.34, 95% CI 0.98 to 1.82, *P* = 0.06), recessive (OR = 1.56, 95% CI 0.50 to 4.84, *P* = 0.44), homozygote (OR = 1.64, 95% CI 0.53 to 5.11, *P* = 0.39), heterozygote (OR = 1.32, 95% CI 0.96 to 1.81, *P* = 0.09) and allelic comparison (OR = 1.32, 95% CI 0.99 to 1.75, *P* = 0.06) models (Table 2; Fig. 4). In subgroup analysis stratified by type of POAG, the meta-analyses indicated no significant association of this SNP with POAG in any of the genetic models in non-HTG. Although two study involving 216 HTG cases and 256 controls showed significant association with POGA risk under dominant (OR = 1.69, 95%CI: 1.07 to 2.12, *P* = 0.03), heterozygote (OR = 1.65, 95% CI: 1.03 to 2.65, *P* = 0.04) and allelic comparison (OR = 1.61, 95% CI: 1.06 to 2.42, *P* = 0.02), there was no statistical significance when Bonferroni correction was considered **(**Table 2 **)**.

Power calculation on the pooled frequencies showed that the statistical powers were all lower than 80% for allele models of the three SNPs (Table 2).

### Sensitivity analysis

**Fig. 5:**
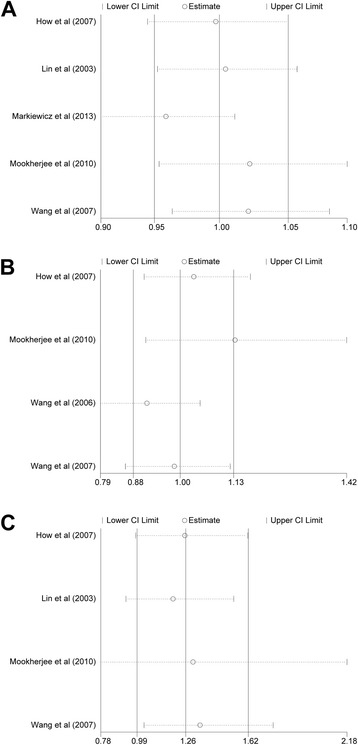
Sensitivity analysis of the correlation the *IL-1* polymorphisms and POAG risk. (A) Sensitivity analysis for the *IL-1β* rs16944. (B) Sensitivity analysis for the IL-1α rs1800587. (C) Sensitivity analysis for the *IL-1β* rs1143634. Abbreviations: POAG, primary open-angle glaucoma; CI, confidence interval

To examine the stability of pooled results, sensitivity analysis was conducted by sequentially excluding individual studies and calculating the pooled ORs for the remaining studies. As shown in Fig. 5, we found no individual study affected the pooled OR in the dominant models of all three SNPs.

### Publication bias

**Fig. 6:**
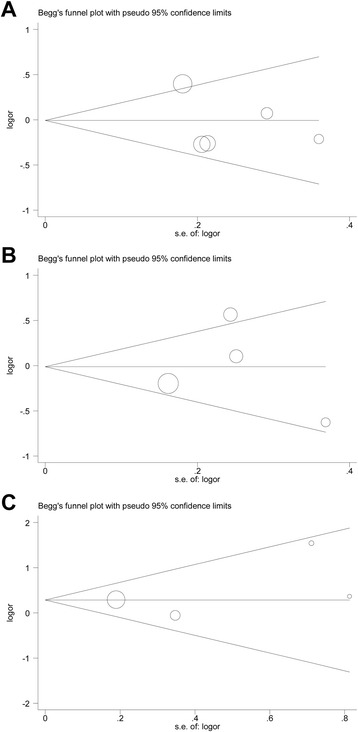
Funnel plots for studies investigating the effect of *IL-1* polymorphisms on POAG risk. (A) Funnel plot for publication bias in the *IL-1β* rs16944. (B) Funnel plot for publication bias in the IL-1α rs1800587. (C) Funnel plot for publication bias in the *IL-1β* rs1143634. Abbreviations: POAG, primary open-angle glaucoma; OR, odds ratio

Funnel plots (Fig. 6) and Egger’s linear regression test found no evidence of publication bias for the dominant models of SNP rs16944 (P_Egger’s_ = 0.57), SNP rs1800587 (P_Egger’s_ = 0.98) and SNP rs1143634 (P_Egger’s_ = 0.56).

## Discussion

This is the first meta-analysis to summarize the evidence of associations between *IL-1* gene cluster polymorphisms and susceptibility of POAG. Our data revealed that the SNPs of *IL-1β* rs16944, *IL-1α* rs1800587 and *IL-1β* rs1143634 were not associated with the POAG risk. Stratification analyses showed that *IL-1β* rs1143634 has a suggestive associated with the risk of HTG, however, this association was feeble after Bonferroni adjustments.

Recently, polymorphisms in the *IL-1* gene clusters have been shown in chronic neurodegenerative disease, such as multiple sclerosis [21], Parkinson’s disease [22] and Alzheimer’s disease [23]. The C allele of rs16944 and rs1800587, and the T allele of rs1143634 has been speculated to influence the risk of POAG by altering the expression level of the respective proteins, because the sites of rs1800587 and rs16944 are located within the transcriptional promoter regions of the *IL-1α* and *IL-1β* genes, respectively, and the site of rs1143634 is within the coding region of the *IL-1β* gene [24, 25]. *IL-1* is a key mediator of immune and inflammatory responses, which act directly to mediate a number of cellular responses. Laboratory studies have showed that *IL-1* could promote ganglion cell loss and optic nerve damage by increasing matrix mellanoproteinase-9 (*MMP-9*) synthesis in experimental models [26, 27]. *IL-1* has also been reported to induce nitric oxide synthesis and reactive oxygen species (ROS), which involved in RGC damage leading to neurodegeneration [28]. However, some studies have put forward argument and their results pointed to certain neuro-protective role of *IL-1* [5, 6]. Therefore, we undertake the present meta-analysis on all the available data to establish a more robust estimate of the association between *IL-1* gene clusters polymorphism and POAG.

Although the meta-analysis for overall studies showed that all the three *IL-1* clusters polymorphisms were not associated with the POAG risk, the results of subgroup analyses should arouse our attention. On the one hand, genetic heterogeneity for POAG may exist in different populations. Stratified analyses in the current meta-analysis based on ethnicity showed that there is no statistical evidence of significant association between the rs16944 and POAG in Asian population. However, one study in Caucasians showed that the SNP rs16944 is important risk factors associated with POAG [7]. Moreover, of the included studies for the Asian population, one studies [7] found a possible association of the SNP rs1143634 with POAG in Taiwan population but this association could not be further replicated in Singaporean [9] Chinese and Indian subjects [12]. Thus, more studies with POAG cohort from different ethnic background are needed to further define this association.

On the other hand, genetic heterogeneity for POAG may exist in different subtypes of this disease. For example, Wang et al. found an increased risk for individuals carrying T allele of *IL-1α* rs1800587 in HTG patients with an IOP > 21 mmHg [8] but not for normal-tension glaucoma (NTG) cases with IOP < 21 mmHg [10]. Similarly, the SNP rs1143634 seem to be associated with HTG rather than NTG. Glaucomatous damage to the retina and optic nerve is often accompanied by pathological IOP elevation. These findings of our meta-analysis suggested that the pathogenesis and effect of *IL-1* may be different between HTG and NTG. Laboratory and clinical studies are needed to further clarify the molecular pathogenesis of the two subtypes of POAG as well as the role of *IL-1* on IOP elevation.

Publication bias should be considered as it is an important factor that affects the reliability of the results of meta-analyses. In the current study, both Begg’s and Egger’s test were used to assess the potential publication bias and failed to detected a significant bias in all genetic models (Fig. 5), demonstrating the robustness and credibility of the present meta-analysis. However, some limitations of this meta-analysis should be taken into careful consideration. Firstly, some unescapable bias may exist in the results as only published studies with full-text were included in our analysis. Secondly, haplotype analysis for the possibility of linkage disequilibrium between SNPs was not performed in the present meta-analysis. How et al. [9] found that The TT haplotype of rs1143634 and rs16944 together and the TTT haplotype of rs16944, rs1800587 and rs1143634 was significantly more common in normal control subjects than in those with POAG. Further investigations of the haplotypic effect of a gene and the study of multiple polymorphisms in different genes are needed. Thirdly, the results of power calculations indicated that the combined sample sizes (overall and subgroups) in the current meta-analyses were still inadequate and underpowered to detect the association of *IL-1* gene SNPs with POAG, due to limited availability of published data. Finally, the strength of association between *IL-1* gene SNPs and POAG was carried out by unadjusted estimate, we didn’t adjust pool results by the factors like the age, gender, disease severity and genotyping procedure.

## Conclusions

In conclusion, this meta-analysis did not demonstrate an association between the *IL-1* SNPs (rs16944, rs1800587 and rs1143634) and the risk of POAG. Nevertheless, this conclusion should be interpreted with caution and well-designed studies with larger cohorts are urgently warranted to verify the present findings as low statistical powers.

## Additional files

Additional file 1: The full details of databases searching terms. (DOCX 36 kb)s (DOCX 36 kb)

Additional file 2: Modified Newcastle-Ottawa Scale for studies of genetic association. (DOCX 16 kb) (DOCX 16 kb)

Additional file 3: Table S1. Characteristics of included studies. (DOC 67 kb) (DOC 67 kb)

Additional file 4: Table S2. Quality of included studies. (DOC 68 kb) (DOC 68 kb)

## Abbreviations

CI: Confidence interval
HTG: High tension glaucoma
HWE: Hardy-Weinberg equilibrium
IL: Interleukin
IOP: Intraocular pressure
MMP: Matrix mellanoproteinase
NTG: Normal tension glaucoma
OR: Odds ratio
POAG: Primary open-angle glaucoma
RGC: Retinal ganglion cells
ROS: Reactive oxygen species
SCI: Science Citation Index
SNP: Single nucleotide polymorphism
