# Supplementary material for: Association of Interleukin-1 gene clusters polymorphisms with primary open-angle glaucoma: a meta-analysis
Source: BMC Ophthalmol. 2017 Nov 28;17:218. doi: 10.1186/s12886-017-0616-y (PMC5704439; doi:10.1186/s12886-017-0616-y)
Supplement: Supplementary file 1 — The full details of databases searching terms. (DOCX 36 kb)s (DOCX 36 kb) [file 12886_2017_616_MOESM1_ESM.docx]

## Additional file 1 Databases searching terms

## (updated to January 1^st^, 2017)

#### Pubmed Database (n=10)

#1：((((((("glaucoma"[MeSH Terms] OR "glaucoma"[All Fields]) OR ("glaucoma, open-angle"[MeSH Terms] OR ("glaucoma"[All Fields] AND "open-angle"[All Fields]) OR "open-angle glaucoma"[All Fields] OR ("open"[All Fields] AND "angle"[All Fields] AND "glaucoma"[All Fields]) OR "open angle glaucoma"[All Fields])) OR poag[All Fields]) OR OAG[All Fields]) OR (intraocular[All Fields] AND ("hypertension"[MeSH Terms] OR "hypertension"[All Fields]))) OR ("low tension glaucoma"[MeSH Terms] OR ("low"[All Fields] AND "tension"[All Fields] AND "glaucoma"[All Fields]) OR "low tension glaucoma"[All Fields] OR ("normal"[All Fields] AND "tension"[All Fields] AND "glaucoma"[All Fields]) OR "normal tension glaucoma"[All Fields])) OR (ocular[All Fields] AND hypertensive[All Fields])) OR OHT[All Fields] -----**64836**

#2：(("interleukin-1"[MeSH Terms] OR "interleukin-1"[All Fields] OR "interleukin 1"[All Fields]) OR ("interleukin-1"[MeSH Terms] OR "interleukin-1"[All Fields] OR "interleukin 1"[All Fields])) OR ("interleukin-1"[MeSH Terms] OR "interleukin-1"[All Fields] OR "il 1"[All Fields])----- **75335**

#3：(((((("polymorphism, genetic"[MeSH Terms] OR ("polymorphism"[All Fields] AND "genetic"[All Fields]) OR "genetic polymorphism"[All Fields] OR "polymorphism"[All Fields]) OR ("polymorphism, genetic"[MeSH Terms] OR ("polymorphism"[All Fields] AND "genetic"[All Fields]) OR "genetic polymorphism"[All Fields] OR ("genetic"[All Fields] AND "polymorphism"[All Fields]))) OR variants[All Fields]) OR ("mutation"[MeSH Terms] OR "mutation"[All Fields])) OR ("mutation"[MeSH Terms] OR "mutation"[All Fields] OR "mutations"[All Fields])) OR ("Socioaffect Neurosci Psychol"[Journal] OR "snp"[All Fields])) OR ("polymorphism, single nucleotide"[MeSH Terms] OR ("polymorphism"[All Fields] AND "single"[All Fields] AND "nucleotide"[All Fields]) OR "single nucleotide polymorphism"[All Fields] OR ("single"[All Fields] AND "nucleotide"[All Fields] AND "polymorphism"[All Fields])) ----- **1195608**

#1 and #2 and #3: -----**10**

#### EMBASE Database (n=21)

('Interleukin 1' OR 'Interleukin-1' OR 'IL-1') AND (' glaucoma' OR 'open angle glaucoma' OR 'POAG' OR' OAG' OR 'intraocular hypertension' OR 'Normal Tension Glaucoma' OR 'ocular hypertensive' OR 'OHT') AND ('polymorphisms' OR 'Genetic Polymorphism' OR 'variants' OR 'variant' OR 'mutation' OR 'mutations' OR 'SNP' OR 'Single Nucleotide Polymorphism') -----**24**

#### Cochrane Central Register of Controlled Trials (CENTRAL) Database (n=0)

#1： MeSH descriptor: [glaucoma] explode all trees-----**2427**

#2： MeSH descriptor: [Polymorphism, Genetic] explode all trees-----**3462**

#3： MeSH descriptor: [Interleukin-1] explode all trees-----**740**

#4：#1 and #2 and #3-----**0**

## Databases searching terms

## (updated to July 15^th^, 2017)

#### (1) Pubmed Database (n=11)

#1：((((((("glaucoma"[MeSH Terms] OR "glaucoma"[All Fields]) OR ("glaucoma, open-angle"[MeSH Terms] OR ("glaucoma"[All Fields] AND "open-angle"[All Fields]) OR "open-angle glaucoma"[All Fields] OR ("open"[All Fields] AND "angle"[All Fields] AND "glaucoma"[All Fields]) OR "open angle glaucoma"[All Fields])) OR poag[All Fields]) OR OAG[All Fields]) OR (intraocular[All Fields] AND ("hypertension"[MeSH Terms] OR "hypertension"[All Fields]))) OR ("low tension glaucoma"[MeSH Terms] OR ("low"[All Fields] AND "tension"[All Fields] AND "glaucoma"[All Fields]) OR "low tension glaucoma"[All Fields] OR ("normal"[All Fields] AND "tension"[All Fields] AND "glaucoma"[All Fields]) OR "normal tension glaucoma"[All Fields])) OR (ocular[All Fields] AND hypertensive[All Fields])) OR OHT[All Fields] -----**66361**

#2：(("interleukin-1"[MeSH Terms] OR "interleukin-1"[All Fields] OR "interleukin 1"[All Fields]) OR ("interleukin-1"[MeSH Terms] OR "interleukin-1"[All Fields] OR "interleukin 1"[All Fields])) OR ("interleukin-1"[MeSH Terms] OR "interleukin-1"[All Fields] OR "il 1"[All Fields])----- **77224**

#3：(((((("polymorphism, genetic"[MeSH Terms] OR ("polymorphism"[All Fields] AND "genetic"[All Fields]) OR "genetic polymorphism"[All Fields] OR "polymorphism"[All Fields]) OR ("polymorphism, genetic"[MeSH Terms] OR ("polymorphism"[All Fields] AND "genetic"[All Fields]) OR "genetic polymorphism"[All Fields] OR ("genetic"[All Fields] AND "polymorphism"[All Fields]))) OR variants[All Fields]) OR ("mutation"[MeSH Terms] OR "mutation"[All Fields])) OR ("mutation"[MeSH Terms] OR "mutation"[All Fields] OR "mutations"[All Fields])) OR ("Socioaffect Neurosci Psychol"[Journal] OR "snp"[All Fields])) OR ("polymorphism, single nucleotide"[MeSH Terms] OR ("polymorphism"[All Fields] AND "single"[All Fields] AND "nucleotide"[All Fields]) OR "single nucleotide polymorphism"[All Fields] OR ("single"[All Fields] AND "nucleotide"[All Fields] AND "polymorphism"[All Fields])) ----- **1227640**

#1 and #2 and #3: -----**10**

#### (2) EMBASE Database (n=22)

('Interleukin 1' OR 'Interleukin-1' OR 'IL-1') AND ('glaucoma' OR 'open angle glaucoma' OR 'POAG' OR 'OAG' OR 'intraocular hypertension' OR 'Normal Tension Glaucoma' OR 'ocular hypertensive' OR 'OHT') AND ('polymorphisms' OR 'Genetic Polymorphism' OR 'variants' OR 'variant' OR 'mutation' OR 'mutations' OR 'SNP' OR 'Single Nucleotide Polymorphism') -----**24**

#### (3) Cochrane Central Register of Controlled Trials (CENTRAL) Database (n=0)

#1： MeSH descriptor: [glaucoma] explode all trees-----**2494**

#2： MeSH descriptor: [Polymorphism, Genetic] explode all trees-----**3579**

#3： MeSH descriptor: [Interleukin-1] explode all trees-----**780**

#4：#1 and #2 and #3-----**0**

## Records identified through database and manual searching

## (n=39)

**(1) Pubmed Database (n=11)**

1: Mookherjee S, Banerjee D, Chakraborty S, Mukhopadhyay I, Sen A, Ray K. Evaluation of the IL1 Gene Cluster Single Nucleotide Polymorphisms in Primary Open-Angle Glaucoma Pathogenesis. Genet Test Mol Biomarkers. 2016 Oct;20(10):633-636.

2: Itakura T, Peters DM, Fini ME. Glaucomatous MYOC mutations activate the IL-1/NF-κB inflammatory stress response and the glaucoma marker SELE in trabecular meshwork cells. Mol Vis. 2015 Sep 17;21:1071-84. eCollection 2015.

3: Choi A, Lao R, Ling-Fung Tang P, Wan E, Mayer W, Bardakjian T, Shaw GM, Kwok PY, Schneider A, Slavotinek A. Novel mutations in PXDN cause microphthalmia and anterior segment dysgenesis. Eur J Hum Genet. 2015 Mar;23(3):337-41.

4: Markiewicz L, Majsterek I, Przybylowska K, Dziki L, Waszczyk M, Gacek M, Kaminska A, Szaflik J, Szaflik JP. Gene polymorphisms of the MMP1, MMP9, MMP12, IL-1β and TIMP1 and the risk of primary open-angle glaucoma. Acta Ophthalmol. 2013 Nov;91(7):e516-23.

5: Sakaguchi T, Irie T, Kawabata R, Yoshida A, Maruyama H, Kawakami H. Optineurin with amyotrophic lateral sclerosis-related mutations abrogates inhibition of interferon regulatory factor-3 activation. Neurosci Lett. 2011 Nov 21;505(3):279-81.

6: Mookherjee S, Banerjee D, Chakraborty S, Banerjee A, Mukhopadhyay I, Sen A, Ray K. Association of IL1A and IL1B loci with primary open angle glaucoma. BMC Med Genet. 2010 Jun 19;11:99.

7: Wang CY, Shen YC, Su CH, Lo FY, Lee SH, Tsai HY, Fan SS. Investigation of the association between interleukin-1beta polymorphism and normal tension glaucoma. Mol Vis. 2007 May 14;13:719-23.

8: Wang CY, Shen YC, Lo FY, Su CH, Lee SH, Tsai HY, Fan SS. Normal tension glaucoma is not associated with the interleukin -1alpha (-889) genetic polymorphism. J Glaucoma. 2007 Mar;16(2):230-3.

9: How AC, Aung T, Chew X, Yong VH, Lim MC, Lee KY, Toh JY, Li Y, Liu J, Vithana EN. Lack of association between interleukin-1 gene cluster polymorphisms and glaucoma in Chinese subjects. Invest Ophthalmol Vis Sci. 2007 May;48(5):2123-6.

10: Wang CY, Shen YC, Lo FY, Su CH, Lee SH, Lin KH, Tsai HY, Kuo NW, Fan SS. Polymorphism in the IL-1alpha (-889) locus associated with elevated risk of primary open angle glaucoma. Mol Vis. 2006 Nov 15;12:1380-5.

11: Lin HJ, Tsai SC, Tsai FJ, Chen WC, Tsai JJ, Hsu CD. Association of interleukin 1beta and receptor antagonist gene polymorphisms with primary open-angle glaucoma. Ophthalmologica. 2003 Sep-Oct;217(5):358-64.

#### (2) EMBASE Database (n=22)

1: Tremosa L., Cole P., Fernández-Forner D., Castro D. Nme digest. Drugs of the Future 2016 41:7 (437-449)

2: Mookherjee S., Banerjee D., Chakraborty S., Mukhopadhyay I., Sen A., Ray K. Evaluation of the IL1 Gene Cluster Single Nucleotide Polymorphisms in Primary Open-Angle Glaucoma Pathogenesis. Genetic Testing and Molecular Biomarkers 2016 20:10 (633-636)

3: Itakura T., Peters D.M., Fini M.E. Glaucomatous MYOC mutations activate the IL-1/NF-κ inflammatory stress response and the glaucoma marker SELE in trabecular meshwork cells. Molecular Vision 2015 21 (1071-1084)

4: Itakura T., Peters D.M., Fini M.E. MYOC involves the NF-κb pathway by regulation of an il1a feedback loop. Investigative Ophthalmology and Visual Science 2015 56:7 (2009)

5: Harrison S.R., McGonagle D.G., McDermott M.F., Savic S. Response to anakinra is effective for the diagnosis and treatment of systemic autoinflammatory disorder of unknown genetic cause. Annals of the Rheumatic Diseases 2015 74 SUPPL. 2 (860)

6: Jeong S., Patel N., Edlund C.K., Hartiala J., Hazelett D.J., Itakura T., Wu P.-C., Avery R.L., Davis J.L., Flynn H.W., Lalwani G., Puliafito C.A., Wafapoor H., Hijikata M., Keicho N., Gao X., Argüeso P., Allayee H., Coetzee G.A., Pletcher M.T., Conti D.V., Schwartz S.G., Eaton A.M., Fini M.E. Identification of a novel mucin gene HCG22 associated with steroid-induced ocular hypertension. Investigative Ophthalmology and Visual Science 2015 56:4 (2737-2748).

7: Agarwal R., Mohanty K., Mishra S., Kumar D., Dada R., Angmo D., Dada T. Genetic screening & oxidative stress analysis in primary open angle glaucoma (POAG). Investigative Ophthalmology and Visual Science 2014 55:13 (2143)

8: de Jesus A.A., Goldbach-Mansky R. Monogenic autoinflammatory diseases: Concept and clinical manifestations. Clinical Immunology 2013 147:3 (155-174)

9: Tarabishy A.B., Hise A.G., Traboulsi E.I. Ocular manifestations of the autoinflammatory syndromes. Ophthalmic Genetics 2012 33:4 (179-186)

10: Sibley C.H., Plass N., Snow J., Wiggs E.A., Brewer C.C., King K.A., Zalewski C., Kim H.J., Bishop R., Hill S., Paul S.M., Kicker P., Phillips Z., Dolan J.G., Widemann B., Jayaprakash N., Pucino F., Stone D.L., Chapelle D., Snyder C., Butman J.A., Wesley R., Goldbach-Mansky R. Sustained response and prevention of damage progression in patients with neonatal-onset multisystem inflammatory disease treated with anakinra: A cohort study to determine three- and five-year outcomes. Arthritis and Rheumatism 2012 64:7 (2375-2386)

11: Sakaguchi T., Irie T., Kawabata R., Yoshida A., Maruyama H., Kawakami H. Optineurin with amyotrophic lateral sclerosis-related mutations abrogates inhibition of interferon regulatory factor-3 activation. Neuroscience Letters 2011 505:3 (279-281)

12: Novartis. Canakinumab. Australian Prescriber 2011 34:2 (55-56)

13: Yu J.R., Leslie K.S. Cryopyrin-associated periodic syndrome: An update on diagnosis and treatment response. Current Allergy and Asthma Reports 2011 11:1 (12-20)

14: Mookherjee S., Banerjee D., Chakraborty S., Banerjee A., Mukhopadhyay I., Sen A., Ray K. Association of IL1A and IL1B loci with primary open angle glaucoma. BMC Medical Genetics 2010 11:1 Article Number 99

15: Rautenstrauss B., Mardin C. Targeting glaucoma beyond intraocular pressure. Expert Review of Ophthalmology 2010 5:2 (217-224)

16: Walsh G.M. Canakinumab for the treatment of cryopyrin-associated periodic syndromes. Drugs of Today 2009 45:10 (731-735)

17: How A.C.S., Aung T., Chew X., Yong V.H.K., Lim M.C.C., Lee K.Y.C., Toh J.-Y., Li Y., Liu J., Vithana E.N. Lack of association between interleukin-1 gene cluster polymorphisms and glaucoma in Chinese subjects. Investigative Ophthalmology and Visual Science 2007 48:5 (2123-2126)

18: Lin H.-J., Tsai S.-C., Tsai F.-J., Chen W.-C., Tsai J.J.P., Hsu C.-D. Association of interleukin 1β and receptor antagonist gene polymorphisms with primary open-angle glaucoma. Ophthalmologica 2003 217:5 (358-364)

19: Wang CY, Shen YC, Su CH, Lo FY, Lee SH, Tsai HY, Fan SS. Investigation of the association between interleukin-1beta polymorphism and normal tension glaucoma. Mol Vis. 2007 May 14;13:719-23.

20: Wang CY, Shen YC, Lo FY, Su CH, Lee SH, Tsai HY, Fan SS. Normal tension glaucoma is not associated with the interleukin -1alpha (-889) genetic polymorphism. J Glaucoma. 2007 Mar;16(2):230-3.

21: Wang CY, Shen YC, Lo FY, Su CH, Lee SH, Lin KH, Tsai HY, Kuo NW, Fan SS. Polymorphism in the IL-1alpha (-889) locus associated with elevated risk of primary open angle glaucoma. Mol Vis. 2006 Nov 15;12:1380-5.

22: Markiewicz L, Majsterek I, Przybylowska K, Dziki L, Waszczyk M, Gacek M, Kaminska A, Szaflik J, Szaflik JP. Gene polymorphisms of the MMP1, MMP9, MMP12, IL-1β and TIMP1 and the risk of primary open-angle glaucoma. Acta Ophthalmol. 2013 Nov;91(7):e516-23.

#### (3) Manual Search (n = 6)

1: Shastry B S. Genetic susceptibility to primary angle closure glaucoma (PACG). Discovery Medicine, 2013, 15(80):17.

2: Smith J.R., Rosenbaum J.T. Intraocular inflammation and systemic immune-mediated diseases. Current Immunology Reviews 2011 7:3 (378-384)

3: T. Aung; A.C. S. How; X.–Y. Chew; V.H. K. Yong; M.C. C. Lim; K.Y. C. Lee; E.N. Vithana. IL1 Gene Cluster Polymorphisms and Risk for Primary Open Angle Glaucoma in Chinese Patients. Investigative Ophthalmology & Visual Science May 2006, Vol.47, 184.

4: M. Fini; C.–Y. Wang; P.J. Farthing–Nayak; D.L. Budenz; L.M. Ventura; M. Polk; A. Venkatramen; M.B. Gorin; J.S. Schuman; Miami–Pittsburgh Glaucoma Genetics Group. Polymorphisms in the IL–1 Gene Cluster Associated With Reduced Risk for Primary Open Angle Glaucoma in Caucasians. Investigative Ophthalmology & Visual Science May 2005, Vol.46, 2369.

5: Oliveira M B, Melo M B D. Avaliação de polimorfismos nos genes IL1A, IL1B e TNFA em pacientes com glaucoma primário de ângulo aberto. Biblioteca Digital Da Unicamp.

6: Indranil M, Antara B, Subhadip C, et al. Association of IL1A and IL1B loci with primary open angle glaucoma. BMC medical genetics, 2010, 11(1):99.
