# Supplementary material for: Association of Interleukin-1 gene clusters polymorphisms with primary open-angle glaucoma: a meta-analysis
Source: BMC Ophthalmol. 2017 Nov 28;17:218. doi: 10.1186/s12886-017-0616-y (PMC5704439; doi:10.1186/s12886-017-0616-y)
Supplement: Supplementary file 2 — Modified Newcastle-Ottawa Scale for studies of genetic association. (DOCX 16 kb) (DOCX 16 kb) [file 12886_2017_616_MOESM2_ESM.docx]

**Additional file 2 Modified Newcastle-Ottawa Scale for case-control studies of genetic association**

Note: A study can be awarded a maximum of one star for each numbered item within the Selection and Exposure categories. A maximum of two stars can be given for Comparability.

**Selection**

1) Is the case definition adequate?

1. yes, with independent validation ★
2. yes, e.g. record linkage or based on self-reports
3. no description

2) Representativeness of the cases

1. consecutive or obviously representative series of cases ★
2. potential for selection biases or not stated

3) Selection of Controls

1. community controls *and* genetic polymorphism of interest in HWE ★
2. hospital controls *and* genetic polymorphism of interest in HWE
3. not community or hospital controls; genetic polymorphism of interest not in HWE
4. no description

4) Definition of Controls

1. no history of disease (endpoint) ★
2. no description of source

**Comparability**

1) Comparability of cases and controls on the basis of the design or analysis

1. cases and controls of homogeneous ethnic descent ★
2. no evidence of population stratification ★
3. no description

**Exposure**

1) Ascertainment of exposure

1. used quality control procedures (e.g. reanalysis of random samples, analysis of samples with a different genotyping method, analysis of replicate samples, sequencing) *and* blinded to phenotype status while genotyping ★
2. used quality control procedures, but no reported blinding
3. no report of quality control procedures or blinding

2) Same method of ascertainment for cases and controls

1. yes ★
2. no

3) Genotyping call rate

1. >99% call rate ★
2. not reported
