# Supplementary material for: Association of Interleukin-1 gene clusters polymorphisms with primary open-angle glaucoma: a meta-analysis
Source: BMC Ophthalmol. 2017 Nov 28;17:218. doi: 10.1186/s12886-017-0616-y (PMC5704439; doi:10.1186/s12886-017-0616-y)
Supplement: Supplementary file 3 — Characteristics of included studies. (DOC 67 kb) (DOC 67 kb) [file 12886_2017_616_MOESM3_ESM.doc]

**Additional file 3 Table S1. Characteristics of included studies**

| ***Study*** | ***Country*** | ***Ethnicity*** | ***Sample size*** | | ***SNP ID*** | ***Conclusion on POAG association*** |
| --- | --- | --- | --- | --- | --- | --- |
|  |  |  | **Cases** | **Controls** |  |  |
| Lin et al/2003[13] | Taiwan | Asian | 58 | 105 | rs16944, rs1143634 | T allele of rs1143634 is associated with POAG |
| How et al/2007[9] | Singapore | Asian | 194 | 79 | rs16944, rs1800587, rs1143634 | No association |
| Wang et al/2007[10] | Taiwan | Asian | 231 | 245 | rs16944, rs1143634 | No association |
| Markiewicz et al/2013[7] | Poland | Caucasian | 255 | 256 | rs16944 | T allele and T/T genotype is associated with POAG |
| Mookherjee et al/2010[8] | India | Asian | 315 | 301 | rs16944, rs1800587, rs1143634 | No association |
| Wang et al/2007[11] | Taiwan | Asian | 162 | 167 | rs1800587 | No association |
| Wang et al/2006[12] | Taiwan | Asian | 156 | 167 | rs1800587 | T allele is associated with POAG |
| Mookherjee et al/2010 [14]* | India | Asian | 392 | 354 | rs17561, rs2856837, rs12612788, rs11676014, rs6706146, rs11680809, rs4849123, rs7596684, rs1143633, rs1143627, rs1143623, rs10169916, rs13008855, rs2723167, rs2708914, rs11674397, rs2708919, rs12472089, rs3811046, rs3811047, rs2708943, rs2708943, rs2723187, rs2708947, rs2723192, rs4387792, rs11689621, rs2723163, rs11676013, rs11676013, rs11690399, rs1562305, rs6542108, rs895497, rs1867828, rs1013477, rs1006122, rs2305150, rs1374280, rs11687786, rs990524, rs7599662, rs1867834, rs12711749, rs12469822, rs4145013, rs3827763, rs6743376, rs11123160, rs12711752, rs10169599, rs13030546, rs6722922, rs11123161, rs4849152, rs1688076, rs13404928, rs4251961, rs2637988, rs408392, rs380092, rs315952 | rs3811046 and rs3811047 were weakly associated with non-HTG cases in discovery cohort, but not sustained in the replication cohort. |

SNP, single-nucleotide polymorphism; POAG, primary open-angle glaucoma, HTG, high tension glaucoma

* All SNPs including in this study were not investigated in other studies, therefore it was excluded from further meta-analysis.
