# Supplementary material for: Association of Interleukin-1 gene clusters polymorphisms with primary open-angle glaucoma: a meta-analysis
Source: BMC Ophthalmol. 2017 Nov 28;17:218. doi: 10.1186/s12886-017-0616-y (PMC5704439; doi:10.1186/s12886-017-0616-y)
Supplement: Supplementary file 4 — Quality of included studies. (DOC 68 kb) (DOC 68 kb) [file 12886_2017_616_MOESM4_ESM.doc]

**Additional file 4 Table S2. Quality of included studies**

| ***Study*** | ***Selection*** | | | | ***Comparability*** | | ***Exposure*** | | | ***Total score*** |
| --- | --- | --- | --- | --- | --- | --- | --- | --- | --- | --- |
|  | **Adequacy of case definition** | **Representativeness**  **of the cases** | **Selection of Controls** | **Definition of Controls** | **Cases and controls of homogeneous ethnic descent** | **Population stratification** | **Ascertainment of exposure** | **Same method of ascertainment for cases and controls** | **Genotyping call rate** |  |
| Lin et al/2003[13] | * | * | * | * | * | * |  | * |  | 7* |
| How et al/2007[9] | * | * | * | * | * | * |  | * |  | 5* |
| Wang et al/2007[10] | * | * |  | * | * | * |  | * |  | 6* |
| Markiewicz et al/2013[7] | * |  |  | * | * | * |  | * |  | 5* |
| Mookherjee et al/2010[8] | * |  |  | * | * | * |  | * |  | 5* |
| Wang et al/2007[11] | * | * |  | * | * | * |  | * |  | 6* |
| Wang et al/2006[12] | * | * |  | * | * | * |  | * |  | 6* |
| Mookherjee et al/2010 [14] | * |  |  | * | * | * |  | * |  | 5* |
